# The ecological genomic basis of salinity adaptation in Tunisian *Medicago truncatula*

**DOI:** 10.1186/1471-2164-15-1160

**Published:** 2014-12-22

**Authors:** Maren L Friesen, Eric JB von Wettberg, Mounawer Badri, Ken S Moriuchi, Fathi Barhoumi, Peter L Chang, Sonia Cuellar-Ortiz, Matilde A Cordeiro, Wendy T Vu, Soumaya Arraouadi, Naceur Djébali, Kais Zribi, Yazid Badri, Stephanie S Porter, Mohammed Elarbi Aouani, Douglas R Cook, Sharon Y Strauss, Sergey V Nuzhdin

**Affiliations:** Section of Molecular and Computational Biology, Department of Biological Sciences, University of Southern California, Los Angeles, CA 90089 USA; Department of Plant Biology, Michigan State University, East Lansing, MI 48824 USA; Department of Biological Sciences, Florida International University, Miami, FL 33199 USA; Kushlan Institute for Tropical Science, Fairchild Tropical Botanic Garden, Coral Gables, FL 33156 USA; Centre of Biotechnology of Borj Cedria, B.P. 901, Hammam-Lif, 2050 Tunisia; Department of Plant Pathology, University of California Davis, Davis, CA 95616 USA; Instituto de Tecnologia Química e Biológica, Oeiras, Portugal; Department of Evolution and Ecology, University of California Davis, Davis, CA 95616 USA; Center for Population Biology, University of California Davis, Davis, California 95616 USA

**Keywords:** Adaptation, Agriculture, Ecological genetics, Population genetics, Abiotic stress

## Abstract

**Background:**

As our world becomes warmer, agriculture is increasingly impacted by rising soil salinity and understanding plant adaptation to salt stress can help enable effective crop breeding. Salt tolerance is a complex plant phenotype and we know little about the pathways utilized by naturally tolerant plants. Legumes are important species in agricultural and natural ecosystems, since they engage in symbiotic nitrogen-fixation, but are especially vulnerable to salinity stress.

**Results:**

Our studies of the model legume *Medicago truncatula* in field and greenhouse settings demonstrate that Tunisian populations are locally adapted to saline soils at the metapopulation level and that saline origin genotypes are less impacted by salt than non-saline origin genotypes; these populations thus likely contain adaptively diverged alleles. Whole genome resequencing of 39 wild accessions reveals ongoing migration and candidate genomic regions that assort non-randomly with soil salinity. Consistent with natural selection acting at these sites, saline alleles are typically rare in the range-wide species' gene pool and are also typically derived relative to the sister species *M. littoralis.* Candidate regions for adaptation contain genes that regulate physiological acclimation to salt stress, such as abscisic acid and jasmonic acid signaling, including a novel salt-tolerance candidate orthologous to the uncharacterized gene *AtCIPK21*. Unexpectedly, these regions also contain biotic stress genes and flowering time pathway genes. We show that flowering time is differentiated between saline and non-saline populations and may allow salt stress escape.

**Conclusions:**

This work nominates multiple potential pathways of adaptation to naturally stressful environments in a model legume. These candidates point to the importance of both tolerance and avoidance in natural legume populations. We have uncovered several promising targets that could be used to breed for enhanced salt tolerance in crop legumes to enhance food security in an era of increasing soil salinization.

**Electronic supplementary material:**

The online version of this article (doi:10.1186/1471-2164-15-1160) contains supplementary material, which is available to authorized users.

## Background

Adaptation of populations to their local environments plays a fundamental role in the maintenance of genetic diversity [1] and is a common ecological pattern in plants [2]. Reciprocal transplant experiments wherein genotypes are planted across environmental gradients remain a primary tool with which to assess local adaptation [3–5]. Studies investigating the genetic basis of local adaptation have been revolutionized by using high-throughput sequencing to pinpoint alleles that assort across ecological gradients against a genomic background mixed by migration [6–10]. This ‘reverse ecology’ approach utilizes genomic variation to nominate traits important to adaptation, in contrast to the forward genetic approach where traits of interest are genetically dissected [11].

There are multiple metrics that are widely used to detect local adaptation in performance data from reciprocal transplant experiments. The "local versus foreign" comparison requires that genotypes from a given habitat type outperform genotypes from a different habitat type in their source habitat, but are outperformed in the alternative habitat. The "home versus away" comparison requires that genotypes perform better in their source habitat type than they do in the alternative habitat type. The local versus foreign metric has been preferred over the home versus away metric because in order for subpopulations (demes) to resist invasion by foreign genotypes they must outperform these non-adapted genotypes [4]. In addition, the home versus away metric can be obscured by differences in habitat quality–if one habitat type is intrinsically more favorable than the other then all genotypes might perform better there, even if there is local adaptation by the local versus foreign metric. Recently, motivated largely by studies of host-parasite local adaptation, a distinct "sympatric versus allopatric" metric has been investigated from a theoretical perspective [5]. This metric recognizes that in addition to habitat quality, the signature of local adaptation can also be obscured by deme genetic quality. Thus, if some demes contain more deleterious alleles than others, they might be outperformed in both habitat types even if they in fact contain locally adapted alleles. The sympatric versus allopatric contrast considers the meta-population level fit between demes and their habitats, and as a consequence it has greater statistical power than the previous two metrics [5]. Importantly, simulations show that locally adaptated alleles can give rise to patterns of performance variation that do not match the local versus foreign metric.

Local adaptation of wild populations has applied importance, since crops tolerant to increasing drought and salinity are needed to sustain agricultural production under future climates [12]. Warming will cause large parts of the world to become drier, which will interact with irrigation to increase soil salinity, and rising sea-levels can infiltrate agricultural land [13]. Currently, approximately 11% of irrigated agricultural land is salinized and soil salinization is a growing problem worldwide, particularly in the United States, China, India, and Pakistan [14]. The strong selective regime imposed by saline habitats is evidenced by specialized halophytic plant species and ecotypes [15]; such natural populations are an important reservoir of adaptive variation that could be useful in breeding future crops. The legume genus *Medicago* contains 83 species whose native distributions surround the Mediterranean basin [16]. *M. sativa* is an important perennial crop and the annuals *M. polymorpha* and *M. truncatula* are cultivated in Australia. *M. truncatula* has been widely adopted as a model species for legume genetics [17]. *M. truncatula* is diploid and self-compatible, with an estimated selfing rate of ~95% [18]. In addition to genome sequences for both *M. truncatula* (~450 Mbp) and congeners [19], there is whole-genome resequencing data of a range-wide 'HapMap' collection [20–22]. *M. truncatula* populations have been collected across ecological gradients in Tunisia [23, 24]; these Tunisian populations occur on and off naturally saline soils and exhibit genetic variation in salinity responses [25, 26]. Previous work using microarray-based genotyping of twelve Tunisian accessions detected a small number of candidate loci that assorted with saline soils [27].

In this study we combine a series of empirical tests of local adpatation to saline soils with whole-genome polymorphism scans in natural Tunisian populations of *M. truncatula*. We measure performance in field gardens, on field soils, and across salt treatments to measure patterns of adaptation and local adaptation and assess traits under selection. We use genomic data to document patterns of migration and recombination. We identify soil-assorting loci that are candidates for adaptation to salinity and compare them to range-wide allele frequencies and to the sister species *M. littoralis*. Finally, we corroborate candidate genes for salinity adaptation using annotated gene functions.

## Results

### Soil salinity in the field has strong negative impacts on plant performance

**Figure 1:**
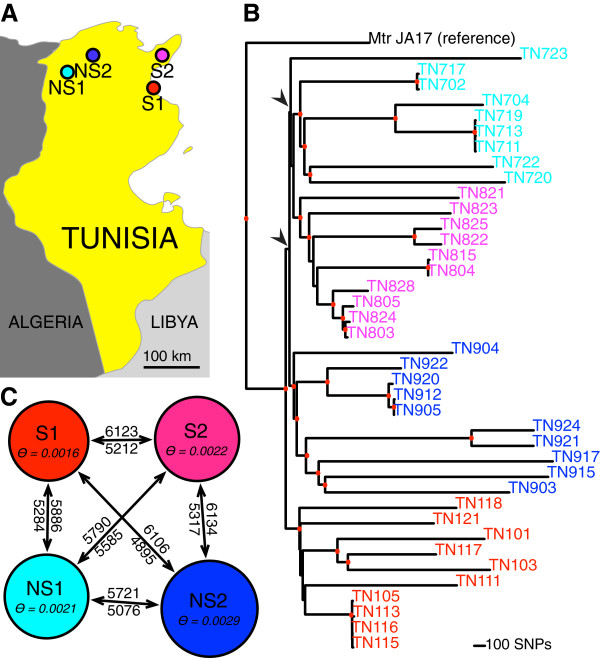
**Tunisian** ***M. truncatula*** **from saline and non-saline sites. (** **A** **)** Tunisian collection sites. **(** **B** **)** Neighbor-joining phylogeny of all SNPs. Red dots indicate nodes with bootstrap support greater than 80%. Nodes subtending splits between populations TN7, TN8, and TN9 are indicated with arrows and do not have bootstrap support. **(** **C** **)** MIGRATE-N analysis of the four populations; numbers above the arrow are *M* estimates going left-to-right and numbers below are the reverse. Results are averaged across ten replicate sets of 96 loci.

**Figure 2:**
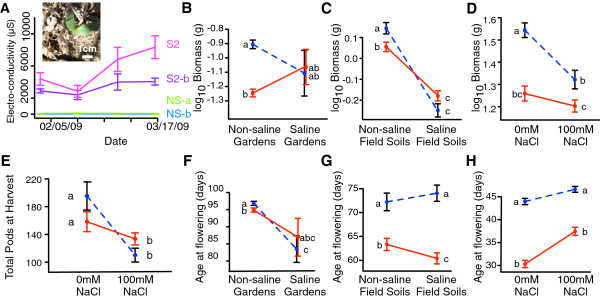
**Saline populations are locally adapted to salinity and flower earlier. (** **A** **)** Soil EC increased in saline field subsites (S2, S2-a) but not in non-saline subsites (NS-a, NS-b). Note that only S2 is an original collection site. (**A** inset) A natural *M. truncatula* germinant emerging from its pod at the beginning of the field experiment (December 2008). **(** **B-H** **)** Phenotypic means and standard errors of saline origin (solid red; S1 & S2) and non-saline origin (dashed blue; NS1 & NS2) genotypes. Plants were grown in saline and non-saline field gardens **(** **B** **,** **F** **)**, field-collected soils in the greenhouse **(** **C** **,** **G** **)**, and 0mM and 100 mM NaCl on a common substrate **(** **D, E, H** **)**. A saline origin by salinity treatment interaction is significant in panels **C**, **D** and **E**.

We assessed the potential for natural selection in saline and non-saline habitats by planting 39 genotypes from replicate saline and non-saline origin sites (Figure 1) into replicate saline and non-saline Tunisian field plots (‘gardens’; Additional file 1). Saline gardens were significantly more saline overall (non-saline field site mean electro-conductivity (EC): 34 μS/m and 43 μS/m; saline mean EC 3,300 μS/m and 5,580 μS/m; *F*_(1, 211)_ = 167.78, *P* < 0.0001; Figure 2, Table 1). EC at saline sites rose during the growing season but remained steady at non-saline sites (Time*salinity of site *F*_(1,1)_ = 9.06, *P* = 0.0029; saline sites: time *F*_(1,1)_ = 9.15, *P* = 0.0031; non-saline sites: time *F*_(1,1)_ = 0.94, *P* = 0.335; Figure 2). In saline gardens, germination was significantly lower than in non-saline gardens (χ^2^_(1)_ = 1,254.7, *P <* 0.001; Additional files 2 and 3); furthermore, within saline gardens the EC of microsites where seedlings failed to emerge was higher than in successful microsites (*F*_(1,113)_ = 13.50, *P =* 0.004). Seedlings that emerged in saline gardens had a 74% lower survival to reproduction than in non-saline gardens (saline: 22.5% vs non-saline: 85.3%, χ^2^_(1)_ = 131.61, *P <* 0.0001, Additional file 2). Thus, natural saline soils pose strong selective challenges in the field to *M. truncatula* through both germination and survival.

**Table 1:** **Soil characteristics**

Soil	Na (meq/L)	SAR	ESP	Na ppm	K ppm	Mg ppm	Ca ppm	pH	HCO3	Organic matter (lbs/acre)	Nitrogen NO3-N ppm
*Field experiment gardens*											
NS-a	2	1	2	28	81	73	2014	7.7	2.6	102	24
NS-b	0.6	0.3	< 0.1	21	81	75	2134	7.8	2.4	63	101
S2-a	49.8	19	21.1	1388	274	593	2199	8.1	2.9	66	26
S2	86.6	14.3	16.6	3684	541	1584	3752	7.8	2	99	14
*Reciprocal soil experiment*											
NS1	0.3	<0.1	1	30	329	157	4257	7.8	2	95	111
NS2	1	0.2	2.5	91	699	158	4550	7.9	3.5	135	48
S1	8.3	9.9	18.5	625	308	624	3302	8.3	2.7	84	46
S2	10.5	12.5	29.8	1291	575	1235	3420	8.0	2.6	99	18

Soil analysis of Tunisian field gardens where field experiment was performed and of field collected soils used in the reciprocal soil transplant experiment. SAR: sodium absorption ratio, ESP: exchangeable sodium percentage.

### *M. truncatula*populations are locally adapted to saline soils at the meta-population level

To measure patterns of adaptation to soil salinity, we conducted a series of field and greenhouse experiments using pod number and aboveground biomass as fitness proxies. *M. truncatula* fruits are barbed pods containing 4–10 seeds and pod number is a relevant fitness metric since pods do not open to disperse seeds. A greenhouse-based pod germination experiment shows that a single seed germinates per pod (averaged across treatments: mean seedlings per pod = 1.30 +/- 0.15 s.d.; no significant effects of soil or salinity) and we observed such germinants alongside our field experiment (Figure 2). It was not possible to grow plants through reproduction in the field, due to concerns regarding genetic contamination of the sites, or in the reciprocal field soil experiment due to space constraints in the Tunisian greenhouse. However, we have two sources of data that enable us to draw conclusions about fitness from biomass data. In greenhouse trials aboveground biomass was significantly correlated with pod production (*r*^*2*^ = 0.32, *P* < 0.0001); furthermore, we found correlations between biomass and pod production for field plants that grew contemporaneously with our experimental plants (saline field gardens: *r*^*2*^ = 0.45, *P* < 0.0001; non-saline field gardens: *r*^*2*^ = 0.41, *P* = 0.0005; Additional file 4). Thus, although imperfect, aboveground biomass is a reasonable fitness proxy for *M. truncatula* at both saline and non-saline field sites and under greenhouse conditions for the range of genotypes included in our experiments.

Our field experiment was designed to assess adaptation to salinity in the presence of natural variation in soils, climate, and biotic interactions. We observed heavy aboveground herbivory in both saline and non-saline gardens [28]. Low germination (120/1240) and survival (23/120) in saline gardens gave poor power to detect whether genotype soil origin affects biomass in saline field gardens (*F*_(1,21)_ = 0.18, *P =* 0.37). However, in non-saline gardens the saline origin genotypes had 2.3 times lower performance than non-saline origin genotypes (*F*_(1,732)_ = 120.54, *P* < 0.0001; Figure 2, Additional files 2 and 3). Lower performance by saline genotypes in non-saline conditions could be due to more deleterious alleles at high frequency in saline populations and/or to the presence of locally adapted alleles that improve fitness in saline habitats but decrease fitness in non-saline habitats.

Definitive evidence of local adaptation to saline soils, defined by the presence of locally adapted alleles segregating across the meta-population, comes from a parallel greenhouse experiment in which seedlings were reciprocally transplanted into raw field-collected soil from the four original collection sites. Plants had lower survival on saline field soils (χ ^2^_(1,606)_ = 31.81, *P <* 0.0001; Additional files 3 and 5), with the lowest survival in the most saline soil (S2 EC = 5.5 mS, 36% survival vs S1 EC = 1.4 mS, 90% survival, *t*_(1,606)_ = 8.31, *P <* 0.0001). As in the field experiment, we assessed performance by aboveground biomass at flowering. Saline-origin genotypes were significantly outperformed by non-saline genotypes on non-saline field soils and tended to outperform non-saline-origin genotypes on saline field soils, with a significant interaction term (*F*_(1,33)_ = 25.01, *P* < 0.0001; Figure 2, Additional files 3 and 5).

While this pattern does not satisfy the strict local versus foreign definition of local adaptation [4], the interaction term between the origin soil type and the destination soil type does indicate that there are segregating alleles that cause saline-origin genotypes to respond differently to saline field soils. We calculated the sympatric versus allopatric contrast following [5] and found it to be both large and statistically significant, despite our study only containing four populations (SA metric = 0.321, *F*_(1,8)_ = 12.9, *P* = 0.00710). Furthermore, we did not detect significant home-soil advantage when considering the performance of genotypes in the field soil from the site at which they were collected (*F*_(4,56)_ = 2.04, *P* = 0.101, Additional file 5); thus, local adaptation appears to be to some shared attribute of saline soil between these populations. To separate the effect of salt from other differences between saline and non-saline soils, we conducted a second greenhouse experiment on a common substrate with added NaCl. Salt treatment caused plants to have 30% lower aboveground biomass and produce 54% fewer pods (Additional file 3). Saline genotypes were smaller than non-saline genotypes under all conditions but showed a smaller decline in biomass between treatments with a significant interaction term (*F*_*(1,36)*_ = 7.7, *P* = 0.0088; Figure 2, Additional file 6). Furthermore, saline-origin plants tended to have more pods under saline conditions while non-saline plants tended to have more pods under non-saline conditions, with a significant interaction term (*F*_(1,36)_ = 9.8, *P* = 0.0034; Figure 2, Additional file 6). Since the sympatric-allopatric contrast requires a reciprocal experimental design at the level of the population to calculate the correct *F*-ratio test [5], we are not able to apply this metric to these data. However, although neither the biomass nor reproduction data satisfy the local versus foreign metric of local adaptation, the significant interaction terms between soil type origin and salt treatment reflects genetic differences between saline populations and non-saline populations in how they respond to salt. We thus infer that saline origin populations contain alleles that mitigate the decline of performance under saline soils, or conversely that non-saline origin populations contain alleles that increase their sensitivity to salinity. We note that non-saline genotypes had higher biomass than saline genotypes under well-fertilized salt treatment in the greenhouse, possibly reflecting pre-adaptations to these high nutrient conditions. However, even in this artificial setting there was a significant interaction between saline origin and salinity for pod production (Figure 2), which we argue indicates the presence of alleles involved in local adaptation to soil salinity.

### Selection analysis and trait differentiation

To gain insight into the underlying causes of salinity adaptation, we used selection analysis to identify traits correlated with differences in reproduction. We detect natural selection on a suite of phenological and morphological traits in the greenhouse—in non-saline environments, traits associated with faster vegetative growth rates are favored by selection, while in saline environments traits associated with salinity tolerance (e.g., increased leaf water content) and earlier germination and flowering are favored (Table 2). Consistent with a response to selection for earlier flowering, saline origin genotypes flower earlier under greenhouse, field soil, and non-saline field conditions (Figure 2, Additional file 3). We note that both of our greenhouse experiments also show genetic variation for flowering time and aboveground biomass but that there were no genotype by treatment interactions after accounting for terms involving soil origin (Additional files 3, 5 and 6).

**Table 2:** **Selection on plant traits under saline and nonsaline treatments**

Trait	S- nonsaline	S- saline	T X E	b- nonsaline	b-saline	T X E
Germ. age	0.215**	-0.006	5.13*	0.024	-0.121*	1.78
1st trifoliate age	-0.113	0.03	0	-0.421*	-0.049	3.49t
1st trifoliate lifespan	-0.018	0.111	1.87	0.072	0.146*	0.29
1st flowering age	0.164*	0.241**	0.93	**-0.072**	-0.195***	3.45t
% early pod set	-0.112	-0.129	0.61	-0.221***	-0.169**	0.34
SLA	0.046	0.038	0.01	-0.07	-0.162**	1.2
Leaflet width	0.077	0.073	0.27	0.207**	0.209***	0
Root length	0.197**	**0.112t**	2.41	0.245***	0.227***	0.12
1-branch length	-0.187**	-0.152*	0.06	-0.077	0.065	3.06t
2nd branch length	**0.172t**	0.201	0.13	0.272***	0.351***	3.18t
3rd branch length	0.161t	-0.023	2.48	0.297***	0.196***	0.6
2nd branch number	-0.042	0.043	0.69	**0.135**	0.273***	2.42
3 branch number	0.075	0.048	0.02	0.373***	0.383***	0.54
Stem diameter	-0.08	**0.049**	2.13	-0.052	0.053	1.16
Root diameter	0.171*	0.005	3.92*	0.264***	0.108t	2.47
Prop. reproduction	0.267***	0.329***	0.09	0.304***	0.346***	0
Root:shoot	-0.035	0.007	0.38	0.181**	0.018	2.85t
LWC	-0.065	-0.116t	1.18	-0.035	-0.172**	2.46
RWC TOP	-0.011	-0.085	0.53	0.166*	0.124*	0.06
RWC Bottom	0.042	0.117*	0.79	0.197**	0.171**	0.01
SWC	**-0.043**	0.027	1.65	0.064	0.057	0.12

Selection differentials (S) and gradients (b) in non-saline and saline treatments, using the fitness metric relative total pod number. SLA: specific leaf area, LWC: leaf water content, RWC: root water content, SWC: stem water content. T X E: test for change in selection between saline and non-saline treatments. Significance is denoted by t: 0.10 > P > 0.05, *P < 0.05, **P < 0.01, ***P < 0.001, ****P < 0.0001. Bold values indicate significance (P < 0.05) with transformed relative total pod number.

### Populations are not structured by soil type

Gene flow could occur among these populations via dispersal movement of spiny pods by livestock transported by truck to pastures across this small country. Whole-genome resequencing of these 39 *M. truncatula* genotypes shows that LD decays to *r*^2^ *<* 0.3 within 10Kb on average, with a high degree of variability around this estimate (Additional file 7). Population subdivision measured by *F*_*ST*_ averages 0.217 genome-wide, but hierarchical analysis shows that this structure occurs largely between populations within soil rather than between saline and non-saline soil types (*F*_*POPULATION/SOIL*_ *=* 0.218, *F*_*SOIL/TOTAL*_ *=* 0.00779, Additional file 8). Pairwise *F*_*ST*_ values are lowest between the two non-saline populations, but the saline populations are as strongly differentiated from one another as they are from non-saline population NS1; the saline populations are equally differentiated from NS2 (Table 3). Analysis of SNPs using STRUCTURE [29, 30] suggests a history of admixture between two ancestral clusters, but these clusters do not track soil habitat (Additional file 9). Despite population structure, divergence among individuals within a population is greater than divergence between populations (Figure 1). Finally, coalescent models implemented in MIGRATE-N [31] demonstrate substantial levels of ongoing migration between all four populations with high estimates in all directions and overlapping confidence intervals for estimates of *theta* (Figure 1, Additional file 10). As there is no evidence of divergence by soil type (Figure 1, Table 3)—replicate saline populations are not more similar to one another at the whole genome level than they are to non-saline populations—these populations enable us to detect islands of genomic differentiation that are candidates for local adaptation to saline soil.

**Table 3:** **Pairwise Fst values between the four populations in this study**

	S1	S2	NS1	NS2
S1	-	0.2968567	0.2940058	0.2625759
S2	(0.2896,0.3054)	-	0.2805411	0.2515398
NS1	(0.2865,0.3023)	(0.2789,0.2891)	-	0.2023075
NS2	(0.2550,0.2701)	(0.2440,0.2582)	(0.1960,0.2099)	-

Means of 100 bootstrap replicates of 10,000 SNPs each, 95% intervals below the diagonal. Saline: S1, S2; non-saline: NS1, NS2.

### Genomic differentiation in relation to salinity in *M. truncatula*

#### Candidate genomic regions for salinity adaptation

**Figure 3:**
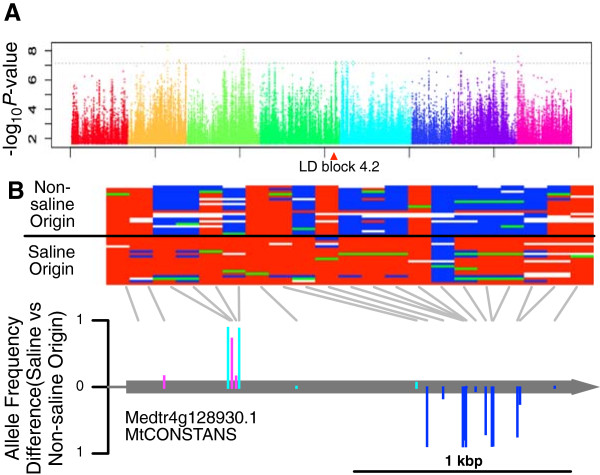
**Genomic regions assorting with saline soil type. (** **A** **)** Soil type assortment *P*-values plotted across the Mt3.5.1 genome. Black dashed line: Bonferroni threshold; triangle: LD block 4.2 that contains Medtr4g128930.1. **(** **B** **)** *AtCONSTANS* ortholog Medtr4g128930.1 showing relationships between Tunisian haplotypes and the gene model. Gray lines connect alleles in the haplotype diagram with their position in the gene model below. Haplotypes—red: saline allele, blue: non-saline allele, green: heterozygous (not included in calculations), empty: no call. SNP bar heights show allele frequency difference between saline and non-saline populations—pink: nonsynonymous, cyan: synonymous, dark blue: UTR.

Our resequencing data identifies loci that may contribute to performance differences among saline and non-saline origin genotypes. Coinciding with meta-population level local adaptation to saline soil, we document soil-type assortment of a small number of genomic regions. Of 677,459 non-singleton SNPs, 40 are significantly associated with soil type after Bonferroni correction (*P* < 0.05/677,459, Figure 3, Additional file 11). These alleles show nearly perfect assortment with soil type; linkage disequilibrium (*r*^2^ > 0.8) circumscribes 16 genomic regions that contain 198 genes (Table 4, Additional file 12). We identify 57 of these as candidate genes with non-synonymous SNPs that assort with soil type; not all of these SNPs were detected in the original scan because of missing data (Table 5, Additional files 13 and 14).

**Table 4:** **Soil-assorting LD blocks**

MtChr	LD block	Number of genes	Starting coordinate	Ending coordinate	Block length (bp)
2	2.1	1	7979654	7981528	1,874
2	2.2	1	23180904	23181977	937
2	2.3	12	29987289	30035909	47,700
3	3.1	67	22518541	22883247	364,706
3	3.2	1	32352422	32363422	11,000
3	3.3	12	33600796	33926116	304,000
4	4.1	1	9036185	9037949	1,764
4	4.2	28	45060331	45166270	85,294
5	5.1	4	1223237	1246052	22,815
5	5.2	3	3806015	3829015	23,000
5	5.3	10	4793100	4837218	44,118
5	5.4	1	7994171	8005200	11,029
6	6	1	10025658	10027422	1,764
7	7.1	2	5878251	5883692	5,441
7	7.2	25	25379197	25519938	140,741
8	8	25	963246	1114570	151,324

LD blocks defined by SNPs that have pairwise correlation > 0.8 and are surrounding the soil-assorting SNPs (Additional file 11).

**Table 5:** **Candidate genes in the soil-assorting genomic regions**

LD block	***M. truncatula***gene ID	Annotation ^†^
2.1	Medtr2g025710.1	Wax biosynthesis; Long-chain-alcohol O-fatty-acyltransferase (Mboat)
3.1	Medtr3g071700.1	Chromatin remodeling; Probable helicase
3.1	Medtr3g071580.1	Phytase; Multiple inositol polyphosphate phosphatase
3.1	Medtr3g071310.1	Protein-protein interaction; Similar to human prot LOC63920 and ZMYM1
3.1	Medtr3g070980.1	Protein turnover; Aspartic proteinase Asp1 (Pepsin A)
3.1	Medtr3g071510.1	Redox regulation; ROS balance; Glutaredoxin
3.1	Medtr3g071480.1	Signaling; Leucine-rich repeat receptor kinase
3.1	Medtr3g071150.1	Vesicle transport; Coatomer subunit beta remote homolog
3.2	Medtr3g098120.1, Medtr3g098140.1	probable FA metabolism; Acyl-coenzyme A thioesterase
3.2	Medtr3g098090.1	Abiotic Stress; Signaling; CPK: Calcium-dependent protein kinase
4.2	Medtr4g128870.1	Abiotic Stress; Carbon metabolism; Trehalose-phosphatase
4.2	Medtr4g128840.1	Carbon metabolism; Xylose isomerase
4.2	Medtr4g128770.1	Biotic stress; Disease resistance; Putative pathogenesis-related protein PR-1-like
4.2	Medtr4g128820.1	Abiotic Stress; Signaling; CIPK Calcium/calmodulin-dependent protein kinase type II
4.2	Medtr4g128930.1	Flowering; Signaling; Constans
4.2	Medtr4g128990.1	Signaling; Serine/threonin kinase-like
5.2	Medtr5g013020.1	Protein membrane localization; Palmitoyl-protein thioesterase 1 precursor
5.3	Medtr5g014910.1	Protein synthesis; Arginyl-tRNA synthetase
5.4	Medtr5g021390.1	Biotic stress/Development; Lignification; Ferulate 5-hydroxylase
6	Medtr6g047210.1	Biotic stress; Disease resistance; NB-LRR protein
7.1	Medtr7g022980.1	Signaling; Zinc finger protein 6
7.2	Medtr7g085120.1	N-fixation; Nucleotide diphosphatase; Apyrase
7.2	Medtr7g084910.1, Medtr7g084940.1	Biotic stress; Phytoalexin biosynthesis; O-acetyltransferase (Flavonoid synthesis)
8.1	Medtr8g008530.1	Electron carrier; Cytochrome P450 (related)
8.1	Medtr8g008500.1, Medtr8g008510.1, Medtr8g008660.1	Biotic/Abiotic Stress; Me-hormone metabolism; Alpha/beta hydrolase Methylesterase
8.1	Medtr8g008640.1	Protein folding; peptidyl-prolyl isomerases (PPIases)
8.1	Medtr8g008680.1	Protein synthesis; Initiation factor eIF-4 gamma like

Genes with non-synonymous mutations in conserved amino acids that assort with soil type. Not included are 5 unknown proteins and 21 hypothetical proteins. Expanded information in Additional files 12, 13, 14, 15 and 16. ^†^Biological Process, Biochemical function, Protein annotation.

We found soil-assorting non-synonymous SNPs in multiple genes annotated with functions that are consistent with adaptation to salinity. In particular, we identify Medtr4g128870.1, a trehalose-6-phosphate phosphatase gene that may be involved in osmotic protection, along with several candidate genes closely related to known regulators of the abscisic acid (ABA) and jasmonic acid (JA) pathways. Medtr3g098090.1 encodes a calcium-dependent protein kinase (CPK) co-orthologous to three Arabidopsis CPKs (*AtCPK4*, *AtCPK11* and *AtCPK12*, Additional file 15) that all regulate ABA signaling (*M. truncatula* contains 23 CPKs compared to 31 in *A. thaliana*). LD block 8.1 contains three paralogs encoding jasmonate methyl esterases that convert methyl jasmonate (MeJA) into biologically active JA; two contain soil-assorting non-synonymous SNPs. Medtr4g128820.1 encodes a CBL interacting protein kinase (CIPK) that is orthologous to *AtCIPK21* (Additional file 15). *M. truncatula* contains 18 CIPKs compared to 26 in *A. thaliana.*

We further identify candidates with roles in phenology regulation and biotic interactions. Saline alleles of Medtr4g128930.1—the ortholog of *CONSTANS*, a central flowering time regulator in *A. thaliana*—differ from non-saline alleles by a non-synonymous SNP (*E155D*) immediately adjacent to the highly conserved B-BOX zinc coordination site (Figure 3). In addition, LD block 7.2 contains four paralogs of *Flowering locus T* (Medtr7g084970.1, Medtr7g085020.1, Medtr7g085030.1, Medtr7g085040.1; Additional file 12). A NB-LRR gene (Medtr6g047210.1) contains 25 soil-assorting non-synonymous changes. Finally, Medtr7g085120.1 is an apyrase-like protein and is in linkage with two genes (Medtr7g084910.1, Medtr7g084940.1) involved in flavonoid synthesis.

The set of SNPs with false-discovery rate (FDR) below 1% contains 4858 SNPs that intersect 573 unique genes (Additional file 16). Among these genes is the only sodium:potassium:chloride symporter annotated in the *M. truncatula* genome (Medtr2g048510.1). Although there was not significant GO enrichment for any of our candidate sets, single genes of large effect could be present in our candidate list without causing GO enrichment.

#### Saline alleles are typically rare across the range and derived relative to an outgroup

Locally adapted alleles that enhance fitness in saline habitats but are deleterious on normal soils are predicted to be rare within the range-wide sample of *M. truncatula*. Furthermore, recent adaptation via these alleles would result in them being derived in comparison to the sister species *M. littoralis.* To test these predictions, which focus on the alleles themselves rather than the populations, we used data from the *Medicago* HapMap project. For each site considered in the soil-assorting test, we calculated the allele frequency across 247 non-Tunisian genotypes of *M. truncatula*. All else being equal, the saline-assorting allele has a 50% chance of being the rare allele in the range-wide sample. However, our Bonferroni-significant saline-assorting alleles were more often the rare allele than expected by chance (27/40, binomial test *P* = 0.038).We additionally ask whether saline alleles tend to be derived relative to an outgroup, the sister species *M. littoralis*. Under neutral evolution we expect a constant rate of fixation of new mutations in a lineage, whereas selection should increase the fixation probability of derived mutations. The *M. truncatula* reference and the *M. littoralis* consensus differed at 117,811 of the 677,459 sites considered in the soil-assorting test, and shared the same allele at 501,289 sites. The saline majority alleles were significantly more often diverged than the reference from the *M. littoralis* consensus (153,000 sites differed, 466,100 sites matched; Fisher's exact test odd's ratio 1.40, *P* < 2.2e-16). Thus, the saline populations’ divergence was used as the background rate to test whether the Bonferroni-significant soil-assorting SNPs were more diverged from *M. littoralis* than expected by chance. Of the 40 soil-assorting SNPs, 23 differ from the *M. truncatula* reference and the *M. littoralis* consensus. Of these, at 3 sites the *M. truncatula* reference and the *M. littoralis* consensus also differ. Saline alleles are more often divergent from both the *M. truncatula* reference and the *M. littoralis* consensus than expected by chance (Fisher's exact test odd's ratio = 3.58, *P* = 0.000152, excluding tri-allelic sites). Thus, saline-assorting alleles are typically rare within *M. truncatula* and derived relative to the sister species *M. littoralis*. Combined, these results further suggest that natural selection in saline habitats has played a role in shaping soil-assorting genomic regions, although differences in demography are a possible alternative hypothesis.

## Discussion

Understanding the maintenance of genetic diversity requires identification of situations in which such genetic variation is adaptive; this problem can be approached from both ecological and genomic perspectives [32]. Local adaptation, as determined by reciprocal transplant experiments, has been found in 71% of published studies [2]. In a growing number of instances, local adaptation has been dissected at the genomic level [6, 7, 10, 21, 33]. In the current study, we integrate manipulative experiments with genomic analyses of saline and nonsaline genotypes from replicate wild Tunisian populations of *M. truncatula* to identify candidate traits and genomic regions associated with local adaptation to soil salinity.

### Ecological signal of local adaptation to saline soils in *M. truncatula*

Combining field and greenhouse experiments, we show that salinity imposes high mortality on *M. truncatula* in the field and that there is a strong signal of local adaptation to salinity at the meta-population level using a reciprocal transplant on field-collected soils. Furthermore, a follow-up experiment manipulating sodium chloride concentrations demonstrates that saline populations are adapted to salt, with non-saline origin genotypes showing greater sensitivity to salinity for both growth and reproduction relative to saline origin genotypes. While none of these experiments are a full reciprocal transplant under field conditions, they nonetheless provide compelling evidence for local adaptation to saline soils and to salinity *per se*.

There is currently debate in the literature regarding how best to identify local adaptation in empirical settings. The strictest definition of local adaptation [4] would require that saline genotypes significantly outperform non-saline genotypes in saline conditions in addition to nonsaline genotypes significantly outperforming saline genotypes in nonsaline conditions (the local versus foreign contrast [2, 34]). Rather, the pattern of fitness variation across saline and non-saline environments that we observe supports that these populations are locally adapted by an evolutionary definition (the sympatric versus allopatric contrast [35, 36]) that considers the meta-population as a whole [5]. Defining local adaptation at the level of a single populations or environments is confounded by habitat quality and/or deme genetic quality, both of which are likely to vary in our study system. The meta-population approach that we take fits models of performance variation that include terms for population, habitat, and soil origin by destination or salt treatment interaction term. An interaction between soil type origin and destination treatment for performance in our experiments reflects a heritable difference between saline and non-saline genotypes in how they respond to saline soils and salt treatments. This interaction is conceptually similar to the 'sympatric versus allopatric contrast' advocated in recent work [5] and we find the sympatric-allopatric contrast metric to be high and significant in our reciprocal soil experiment. Altogether, we conclude that these populations are locally adapted to salinity at the meta-population level and contain alleles with habitat-specific fitness effects.

Under greenhouse conditions, we found the somewhat puzzling result that non-saline genotypes produced greater aboveground biomass than saline genotypes under salt treatment. Several factors may explain this result. First, aboveground growth may not correspond directly with fitness (number of pods) and thus might not reflect adaptedness of populations but rather, different allocation patterns. Indeed, while our experiments show that biomass is a reasonable fitness proxy, the *R*^2^ under greenhouse conditions is only 0.32. Second, if non-saline genotypes have higher fitness due to greater genetic quality of these demes, rather than possession of saline-adapted alleles, migrants from non-saline populations could invade saline populations. However, recombination would move locally-adapted saline alleles into the superior genetic background of non-saline genotypes to create even fitter genotypes. Thus, as long as recombination occurs sufficiently often relative to migration of non-adapted alleles in high quality genetic backgrounds, locally adapted alleles could persist in these populations.

### Evidence for migration and recombination

In order to detect alleles that segregate with saline habitats, outcrossing and migration must occur at appreciable levels. Since *M. truncatula* is highly selfing [18], linkage disequilibrium is expected to be large. However, although we observe higher LD in our study populations (~10 Kbp) than range-wide estimates (~3 Kbp) [20], this still yields gene-scale resolution on average; these patterns closely match our previous microarray-based study [27]. Coalescent-based analysis of migration using the SNPs in this study detected evidence of substantial migration between all pairs of populations, despite the moderate genome-wide levels of population structure as reflected by *F*_*ST*_. Importantly, hierarchical *F*_*ST*_ analysis found that most structuring occurs between populations rather than between soil types. We expect that migration occurs largely by migration of seed pods on the fur of grazing animals such as sheep, which have been moved widely around Tunisia since historic times.

### Reverse ecology gives insight into the mechanisms of salinity tolerance

Genomic analysis identifies a small number of candidate genomic regions for the adaptation to saline soils that we observe at the phenotypic level. Comparison with a range-wide sample shows that saline alleles are typically rare within *M. trunctula* and comparison with the sister species *M. littoralis* shows that saline alleles are typically evolutionarily derived. Both of these patterns could be explained by either stronger genetic drift in saline populations or selection acting on these alleles in a habitat-specific manner. While our coalescent-based analysis does not support differences in effective population size between saline and non-saline habitats, additional data tracking the census size or estimating *N*_*e*_, as has been done previously in other *M. truncatula* populations [37], via time-series analyses would provide power to more rigorously test this alternative hypothesis. Consideration of the functional annotation of soil-assorting genomic regions points towards plausible candidates for locally adapted alleles and nominates multiple pathways and potential selective agents underlying salinity adaptation in *M. truncatula*. In particular, we find soil-assorting candidate genes that are potentially involved in abiotic stress tolerance, stress avoidance through shifts in flowering time, and biotic interactions.

#### Candidates for abiotic stress tolerance

We identify several candidates for adaptation that likely play direct roles in enabling plants to tolerate the abiotic stresses of salinity, which include ion toxicity as well as osmotic stress [38]. Hormone signaling, especially ABA, often mediates acclimation to abiotic stress [39] and multiple candidate genes are closely related to known ABA pathway regulators. First, Medtr3g098090.1 codes for a CPK whose three homologs in *A. thaliana* are all involved in ABA signalling. *AtCPK12* negatively regulates ABA signaling during seed germination and post germination growth [40], while *AtCPK4* and *AtCPK11* are induced by drought and salinity stress and positively regulate ABA-responses and stress tolerance. In addition to impaired ABA signaling, *AtCPK4* and *AtCPK11* mutants are pleiotropic for seedling insensitivity to salt stress and they are impaired in ABA-induced stomatal movement—a process strongly controlled in response to stress. A related experiment found that seedlings of saline origin genotypes from these populations are more sensitive to ABA than non-saline genotypes [41].

Second, the plant hormone MeJA controls stomatal closure and three paralogs encoding the final enzymatic step in this pathway assort with saline soils. The JA pathway is also involved in interactions with herbivores [42]. Third, Medtr4g128820.1 is orthologous to *AtCIPK21*, which is not currently characterized. However, CIPK proteins are known for their roles in integrating environmental cues, especially abiotic stress [43], with roles in salinity tolerance via Na + homeostasis, K+ uptake and guard cell function during dehydration, and regulation of ABA signaling; a novel CIPK protein *HbCIPK2* in the halophyte *Hordeum brevisubulatum* was recently shown to complement salt sensitivity in *A. thaliana* [44]. Together, these facts nominate Medtr4g128820.1 (*AtCIPK21*) as a strong novel candidate for salt tolerance.

Finally, metabolic tolerance to osmotic stress can be achieved by producing small metabolites [38]. Our candidate gene Medtr4g128870.1 encodes trehalose-6-pentose phosphotase (TPP), which converts trehalose-6-phosphate to trehalose. Overexpression of TPP in rice enhances tolerance to both salinity and cold stress [45] and in *M. truncatula* the accumulation of trehalose in nodules enhances salinity stress [46, 47]. We did not find candidates with clear annotations for salt exclusion, unlike in *A. thaliana* where the sodium transporter HKT1;1 controls sodium accumulation in cells and variant alleles of this gene both assort with saline soils and confer increased fitness under salt treatment [48].

#### Early flowering as an avoidance strategy in saline populations

Our study documents both ecological and genomic lines of evidence for the importance of salt stress avoidance through early flowering. A consistently differentiated trait between these populations is flowering time—saline origin genotypes flower earlier in non-saline field conditions, on all field-collected soils, and under both saline and non-saline treatments. Using a greenhouse selection analysis, we detect direct selection for earlier flowering under saline treatment despite an overall selection differential that favors later flowering in both saline and non-saline conditions. Flowering time has been found to play a key role in local adaptation in multiple annual plants [32, 49, 50] and emerges as a candidate mechanism of adaptation from our genomic analyses as well.

We find two separate candidate intervals that likely play important roles in regulating flowering time. One intriguing candidate gene in our study is Medtr4g128930.1, the *M. truncatula* ortholog of *A. thaliana CONSTANS* (*CO*), which contains a saline-assorting nonsynonymous SNP that changes a highly conserved amino acid. This gene encodes a zinc-finger transcription activator that controls the expression of floral-inductive genes, including the transcription factor *Flowering locus T* (*FT*), in a light-dependent manner [51]. A separate candidate genomic region on chromosome 7 contains four paralogs of *FT*, including both *FTa1, FTa2* (currently represented by two genes)*,* and *FTc* but not *FTb1* or *FTb2* (following nomenclature in [52]). Previous work in mapping populations of *M. truncatula* identified a major QTL on chromosome 7 that co-localizes with our region 7.2 and contains all of the *FT* genes in our candidate interval (*FTa1*, Medtr7g084970; *FTa2*, Medtr7g085020 and Medtr7g085030; *FTc*, Medtr7g085040) in addition to a homolog of *CONSTANS* (Medtr7g083540) [53, 54]. Complementation tests of the late-flowering *ft-1 A. thaliana* mutant determined that *MtFTa1* and *MtFTc* are both functional copies of *FT* that regulate flowering time [52]. Additional work studying a *Tnt1* insertion mutant with earlier flowering connected this phenotype to Medtr7g084970 (*FTa1*) and concluded that the proposed *MtCO* (Medtr7g083540) was not orthologous to *AtCO* [55]; our reciprocal BLAST identifies Medtr4g128930.1 as the true ortholog to *AtCO*. Finally, transcription of *FTa1* in *M. truncatula* has been shown to accumulate in leaves during development and respond to vernalization and day-length in tandem with flowering time, while overexpression promotes earlier flowering and *Tnt1* mutants of *MtFTa1* are late flowering [52].

To our knowledge, allelic variation in *CONSTANS* or *FT* have not been previously linked to abiotic stress tolerance. The seasonal rise in salinity at our field sites may select for early flowering to avoid salt. We detect a significant selection gradient favoring earlier flowering in our greenhouse experiment, but only under salt treatment. Coupling these results with our finding that saline genotypes flower much earlier than non-saline genotypes across a suite of conditions, we conclude that flowering time likely plays a major role in adaptation to saline soils. In *A. thaliana,* the genomic basis of flowering time and its adaptive value have been documented in detail through of decades of forward genetics, genome-wide association studies, and ecological analyses [32, 33]. Avoidance of stress is a common strategy in annuals [56] and early flowering is a common breeding target.

#### Candidates linked to biotic interactions

Adaptation to contrasting environments may occur in response to multiple selective agents simultaneously, including antagonists and mutualists. Furthermore, biotic interactions may exert natural selection in a manner that depends upon the abiotic context, for example, saline soils can modulate disease pressure in tomato [57]. At the molecular level, biotic and abiotic stress factors often interact, with JA and ABA playing additional roles as biotic stress signals [58]. In our field experiment, we observed heavy herbivory in both saline and non-saline gardens [28], particularly by *Hypera* weevils in non-saline sites; thus biotic interactions could be additional sources of selection that differ across habitats.

The NB-LRR gene Medtr6g047210.1 falls on chr06 and contains 25 soil-assorting nonsynonymous changes. NB-LRR genes regulate disease resistance [59] and most of these SNPs affect the leucine-rich repeat domain that confers pathogen specificity and is under positive selection in other NB-LRR proteins [60]. In addition, the candidate genes Medtr7g084910.1 and Medtr7g084940.1 are homologous to the *A. thaliana* T8F5.23 protein, which is predicted to act in the flavonoid pathway to produce phytoalexins. Finally, the apyrase candidate gene (Medtr7g085120.1) is strongly expressed during early nodule development [61] and in roots under salt stress [62]. Thus, while it may play a role in symbiotic nitrogen-fixation it may also respond to stress directly. Recent work using the range-wide collection of *M. truncatula* identified three candidate loci associated with climatic variables which have annotated roles in biotic stress, including one also found in a range-wide *A. thaliana* study [21, 33]. However, we do not detect differentiation at these previously identified loci in the present, locally focused study.

### Limitations of the current study

The NB-LRR gene Medtr6g047210.1 that is differentiated between saline and non-saline populations represents the sole candidate gene in common with our previous study [27]. Given that the vast majority (33/39) of genotypes in the current study differ, with only three out of four populations in common, it is not surprising that we obtained largely different soil-assorting regions. Larger population sizes and additional geographically disjunct saline populations are needed to assess the repeatability of the candidates identified here.

Additional work explicitly manipulating rhizobial symbionts and antagonists, including herbivores and pathogens, will be required to determine whether these partners play a role in adaptation to salinity in *M. trunactula*. Furthermore, the field and greenhouse experiments reported here were done without manipulating competitive environment or maternal environment. A related experiment with a subset of the same *M. truncatula* genotypes found that phenotypic responses to salinity depend upon both the presence of a conspecific competitor and upon whether the maternal environment was saline or non-saline [28].

As the candidate genes and pathways discussed above remain to be validated with functional genomic tools, there are several non-adaptive alternative hypotheses for the differentiation that we observe. First and foremost, local adaptation may be caused by fixation of habitat-specific deleterious alleles through drift [63]. Differentiated loci may also occur through gene surfing during population expansion or through segregating incompatibilities [64]. The parallel genomic patterns in our two saline populations argue against such processes, but this inference would be greatly strengthened by the inclusion of additional saline and non-saline populations. A quantitative genetic approach involving between-population crosses would further strengthen the connection between genomic variation and adaptive phenotypic variation.

While we have focused on mutations that alter the amino acid sequence of proteins, we note that expression variation could play an equally important role in adaptive evolution [65]. Annotating regulatory regions within the *M. truncatula* genome will elucidate the potential role of non-coding sequence polymorphism. Future experiments assaying gene expression would yield further insight into the molecular pathways underlying adaptation to saline soils.

## Conclusions

Integrating genome scans with ecological experiments and selection analysis in the model legume *Medicago truncatula* identifies mechanisms by which legumes may have adapted to saline soils. Importantly, while the reverse ecology approach that we take does not enable us to connect genomic candidates to salinity adaptation directly, these candidate genes elucidate potential routes to adaptation in natural contexts where soil salinity may be consistently associated with variation in other abiotic and biotic factors. The diversity of implicated processes underscores that complex field environments can impose selection on multiple correlated traits.

Elucidating the genetic basis of adaptation to saline soils provides insights for breeding improved abiotic stress tolerance in crops and informs efforts to protect species from consequences of climate change such as sea level rise and coastal salinization. In total, our results point to the importance of both salt tolerance and salt avoidance. We have uncovered several promising targets with roles in abiotic stress tolerance and early flowering time, which require further validation but could ultimately be used in breeding salt tolerance in crop legumes to ensure food security in an era of global change.

## Methods

### Materials

We focused on four populations in northern Tunisia (Figure 1) with similar climate but differing soil salinity. Two saline populations, S1 (Enfidha, TN1 in [23, 24]) and S2 (Soliman, TN8), come from coastal ‘*sebkha*’ sites with heavy clay soils. Two non-saline populations, NS1 (El Kef, TN7) and NS2 (Bulla Regia, TN9), originate from loam agricultural soil and clay loam soil. Phenotypic and genomic analyses were conducted on 39 selfed genotypes—10 originating from S1, S2 and NS2 and 9 originating from NS1.

### Phenotypic experiments testing for adaptation to soil salinity

Field and greenhouse experiments with all 39 sequencued genotypes were conducted to measure performance and identify traits associated with salinity adaptation. In the field, a common garden experiment was conducted across replicated saline and non-saline environments in Tunisia. In the greenhouse, we conducted two experiments—one using saline and non-saline field soils in Tunisia and the other manipulating only salinity in a standardized substrate.

#### Field experiment

To quantify performance of the sequenced *M. truncatula* genotypes in nature, seeds were sown into saline and non-saline sites near the Center for Biotechnology Borj Cedria (CBBC), Tunisia. Fifteen scarified seeds of each of the 39 sequenced genotypes were planted in a fully randomized design in two replicate saline and non-saline field sites (‘gardens’) that were fenced to prevent grazing (soil analysis in Table 1). Gardens were located where *M. truncatula* plants were observed in previous years and in saline gardens seeds were planted around halophytic shrubs to provide suitable microsites. Germination, survival, herbivory, and flowering were recorded throughout the experiment. To prevent genetic contamination*,* plants were harvested at flowering and aboveground biomass was used as a fitness proxy.

#### Reciprocal soil experiment

In lieu of a field reciprocal transplant, which was impossible due to the lack of protected areas, we reciprocally planted all genotypes into field-collected soils from each of the four original collection sites (S1, S2, NS1, NS2). Soil from each site was homogenized and placed in pots at the CBBC greenhouse, Tunisia. Six replicate surface-sterilized, pre-germinated seedlings per genotype were transplanted into each soil; seedlings that died within ten days were replaced. Plants were watered with distilled water and flowering and aboveground biomass were recorded. Three months into the experiment the greenhouse cooling system broke, so only the 658 individuals harvested prior to the failure were used for analyses. These represented all treatment and source population combinations, but the earlier flowering saline populations were over-represented.

#### Greenhouse NaCl experiment

To quantify adaptation to NaCl, a second greenhouse experiment was conducted in which scarified seeds were planted in sterile horticultural sand in 656 ml DeePots in a UC Davis greenhouse, USA, with 0 or 100 mM NaCl treatments starting two weeks after planting. Plants were fertilized twice a week with 100 ml Fahräeus nutrient solution supplemented with 3mM KNO_3_. Water was gradually reduced after 3 months to simulate Mediterranean precipitation, then stopped after 4 months. Plants were grown through fruiting and a suite of traits were measured (see below).

### Phenotypic data analysis

Across our three main experiments we analysed variation in germination, survival, flowering time, and performance in relation to the main effects and interactions between origin soil type, source population nested within origin soil type, genotype, destination soil type or salinity treatment, and plot within soil type (for field and field soil experiments). Germination and survival to reproduction were analyzed as binary traits using PROC GLIMMIX or GENMOD and continuous traits were analyzed using PROC MIXED or PROC GLM with SAS (Version 8.1 or 9.2; SAS Institute Inc., Cary, NC, USA). Terms containing genotype were considered random effects while other factors and interactions were considered fixed. PROC GENMOD and GLM were used for the field data because we could not fit genotype terms since all replicates of some genotypes died in the saline gardens. Significance of random effects are χ ^2^ tests comparing the difference in -2 log likelihood of the full model and the model excluding that factor with 1 degree of freedom. Differences between soil type origin by salinity destination combinations were tested using Tukey post-hoc tests.

### Traits measured in greenhouse NaCl experiment

We measured a suite of traits documenting phenology (dates of germination, first leaf emergence, second leaf emergence, third leaf emergence, cotyledon death, first leaf death, second leaf death, first flowering, first fruit, death), vegetative growth (leaf number censused five times, number of primary, secondary and tertiary branches, length of primary, secondary and tertiary branches, biomass), functional traits (specific leaf area, root length, root diameter, stem diameter), water content (leaf, root, and stem) and reproductive output (early pod number, total pod number, proportion of biomass allocated to reproduction).

### Selection analysis

To identify traits associated with salinity adaptation we performed phenotypic selection analyses [66] on the greenhouse NaCl experiment using total pod number as the fitness metric. Fitness was relativized and trait values standardized [67]. Direct selection was calculated by partial regression coefficients (*b*) from multiple regression of all traits on relative fitness (PROC REG); to reduce collinearity variance inflation factors were required to be less than 3.7. Total selection (*S*) was calculated using linear regression of each trait on relative fitness. ANCOVA tested differences in selection between treatments.

### Field data on pod production

Simultaneously with our field trial, we identified naturally occurring plants within our field exclosures and followed them throughout their lifespan to determine relationships between size and age at first flowering, biomass, and lifetime pod production. Pods were dabbed with paint so that any dropped from the plant could be recovered. After senescence shoot and root biomass was measured and pods were counted and weighed.

To determine the relationship between biomass and total pod production in naturally occurring plants within field exclosures, we used multiple regression to determine the relationship of size traits (i.e. stem length, number of leaves, and dry biomass) and phenology (days to first flower) to total pod production within each planting soil type (saline or non-saline). Data were transformed to help meet assumptions of normality and heteroscedasticity. We also examined the correlation between total pod number and all morphological and phenological traits.

### Pod germination experiment

To test the hypothesis that pod number is a relevant fitness metric, we planted seeds and pods from five USDA lines of *M. truncatula* into soil with and without salt treatment. We planted five USDA lines (Italy W6 6021, Morocco 2653, Morocco 2647, Morocco CPI 135030, Portugal EMP 3173) as pods or seeds into UCD mix soil (2:1 Potting soil: horticultural sand) or soil collected from a creek bed at Putah Creek Park in Northern Davis, California. Seeds/pods were watered with 0mM or 100 mM NaCl mixed with UCD fertilizer water every other day for the 70 day experiment. Germination data was analyzed using PROC GLIMMIX.

### Field data on soil salinity and electro-conductivity

Five soil samples per garden were analyzed for salinity and nutrients (A&L Western Agricultural Laboratories, Modesto, California). Soil electro-conductivity was measured biweekly with a hand-held probe at 5 points located in each of the four gardens (Spectrum Technologies, Plainfield, Illinois). During the plant harvest (i.e., at flowering), we measured electro-conductivity of microsites located within the saline gardens for 57 microsites where seedlings emerged and 57 microsites where seedlings failed to emerge (114 total).

Electro-conductivity across sites and time-points from the field experiment was analyzed using repeated-measures ANCOVA with sites nested within soil type and temperature as a covariate; separate models were fit for saline and non-saline gardens. Electro-conductivity of microsites where seedlings emerged or failed to emerge was compared using a t-test.

### Sequencing methods

Aseptically-grown root tissue was used to construct Illumina libraries for whole-genome sequencing [68]. Genomic DNA was isolated with QIAGEN Plant DNeasy (Qiagen USA, Gaithersville, MD), then 5 μg of DNA in 200 μl nuclease free water was fragmented by sonication (Branson Sonifier 250) using: Duty cycle - 80%, Output Control - 1.8, 4 rounds of 20 pulses, with samples cooled on ice between rounds. Fragments were size selected (200–400 bp) on a 1.5% agarose gel, blunt-end repaired using Epicentre End-it Repair Kit, and an A added with NEB Klenow 3' to 5' exonuclease. Illumina adaptors were ligated using Epicentre Fast-Link DNA Ligation Kit and libraries size selected (250–550 bps) on a 1.5% agarose gel, then enriched using NEB Phusion polymerase with PE primers 1.0, multiplexing PCR primers and Illumina indexes. Libraries were quantified using Invitrogen Qubit and sequenced at NCGR (Santa Fe, NM) in 90bp paired-end format on an Illumina GAIIx with ~6x sequencing depth per genotype.

To estimate the rate of false positives in our SNP-calling, we performed Sanger sequencing on three candidate loci (see below) and one conserved locus: COS6 (F - GTGGAAGGCACCATTGATTGACAAC; R - TCTTCTTCTCAGCCTCTTCAAATGC), I3C4 (F - AACGTGGAAAATGAATCGTACC; R - TCAACTATTTGTTGGTCCTTGC), G2C3, (F -TGTAACACTTTCACCTCACTGC; R - TCTGGAGCTGGGATAAACTCC), and G1C2 (F - AGCGAATCGAAATTAACTAGGC; R - ACCCTAGCAACATGGTAC ACG). Traces were assembled in CodonCode 2.0.6 (CodonCode Corporation, Dedham, MA).

### SNP-calling and validation

Reads were uniquely mapped to the genome assembly Mt3.5.1, a genome assembly of 246 Mbp that spans a physical distance of 375 Mbp with 47,845 supported genes capturing ~94% of expressed genes [17], using BWA 0.5.7 [69] with <8 mismatches. While our criteria allowed up to 9% divergence, some of these mismatches could have arisen from errors associated with base-calling. We thus opted to accommodate different divergences between our samples, since the *M. truncatula* reference genome was from a different population. Furthermore, we utilized only polymorphisms that were based upon multiple uniquely mapped reads, thereby reducing potential errors associated with allowing a high number of mismatches. SNPs were called with the GATK Unified Genotyper [70] in a population aware manner using -stand_call_conf 30.0 and -stand_emit_conf 10.0, with <2,000 read depth, requiring calls in > =30 lines. Statistics for regions with Sanger data are plotted in Additional file 17. Requiring ‘*QD*’, the variant quality divided by the depth of non-reference calls, to be >30 resulted in >95% of polymorphic sites called using the Illumina sequencing data to be ‘true positives’ based on the Sanger data (85 Illumina calls with *QD* >30, 81 polymorphic in Sanger, false positive SNP rate 4.9%, Additional file 18). True and false positives were not related to allele frequency. Sanger data, masked for coverage by the Illumina reads, contained 95 SNPs, so our method called 85.26% of true polymorphic sites. We detected a total of 2,446,817 SNPs with QD >30 called in > =30 individuals. For annotation, filtering criteria were relaxed to require calls in 20 lines, resulting in 4,004,348 polymorphic positions with functional annotations using SNPEff (http://snpeff.sourceforge.net/) with Mt3.5.1 IMGAG gene predictions. Residual heterozygosity ranged from 3-13%. Requiring sites be homozygous, biallelic, and called in at least 7 individuals per population resulted in 886,770 SNPs, with 677,459 non-singleton SNPs; this set of SNPs was utilized in population genomic analyses. Downstream analyses were done in R (version 2.6.2 or 2.11.1, [71]).

### Population genomic analyses

#### Population structure and migration

We used 17,353 SNPs that were called in all 39 genotypes for STRUCTURE [29] analysis, varying *k* from 1 to 8 with five replicate runs. We used delta K to determine the most likely value of *k* [30]. We computed hierarchical and pairwise F-statistics for each polymorphic site in the genome using ‘hierfstat’ [72].

We used MIGRATE-N [31] to assess patterns of ongoing migration among these populations, with analysis of ten different replicate sets of 96 10Kbp loci. Each Bayesian run had a burn-in of 10,000,000 to ensure stationarity, four chains with temperatures 1.00, 1.50, 3.00, and 1e + 06, and was run for 1,000,000 steps with samples every 100 steps. The starting genealogy was the UPGMA tree and starting values were calculated using *F*_*ST*_; *theta* priors were Uniform(0, 0.5) and *M* priors were Uniform(0, 1e + 04). A full model was fit, with each parameter allowed to vary. Run performance was assessed through the acceptance ratio and effective sample size.

#### Soil-assorting candidate loci

For non-singleton sites, we computed a chi-squared test of allele frequencies in saline versus non-saline populations, then converted *p*-values to false-discovery rate *q*-values [73]. LD blocks around soil-assorting SNPs were defined by *r*^2^ > 0.8. Genes with non-synonymous SNPs that assort with soil type (allele frequency difference > 0.7) had Mt3.5.1 annotations confirmed using the structural phylogenomic encyclopedia PhyloFacts [74] and the orthology group prediction tool PHOG [75]. Protein alignments containing sequences from numerous angiosperms were used to identify conserved amino acid positions. For selected candidate genes, we identified *M. truncatula* and *Arabidopsis thaliana* homologs using BLAST with a relaxed E-value (1e-30), then constructed a maximum likelihood tree to identify protein subfamilies. Protein alignments using MAFFT and Maximum Likelihood analyses using RAxML Black Box and the JTT substitution matrix were conducted through the CIPRES portal (http://www.phylo.org/).

#### Comparison of soil-assorting loci to range-wide (non-saline origin) allelic frequencies and divergence relative to the sister species

If *M. truncatula* has recently adapted to saline habitats, we predict that saline-associated alleles will be rare in the range-wide HapMap samples that originate exclusively from non-saline habitats. *Medicago* HapMap SNPs in Mt3.5.1 frozen June 6, 2012 were downloaded from the UMN server and 247 *M. truncatula* genotypes not originating from Tunisia were compared to our genotypes. Frequency distributions of probabilities were compared for synonymous and non-synonymous sites between saline and non-saline subgroups using the KS test. Fisher’s exact test assessed whether the number of significantly deviating SNPs was consistent with the null expectation of equality between saline and non-saline populations.

To determine whether saline-associated alleles are evolutionarily derived, we compared them to the sister species *M. littoralis.* Raw Illumina sequences for three *M. littoralis* lines (HM017, HM029, and HM030) were downloaded from ftp.ddbj.nig.ac.jp and aligned to the Mt3.5.1 reference as above. SNPs were called using GATK and the majority base taken as consensus.

### Availability of supporting data

The data sets supporting the results of this article are available in: the Sequence Read Archive: accessions SRA020975 and SRA026748; and Genbank: accessions JX502023-JX502175.

## Electronic supplementary material

Additional file 1: **Map of field experimental sites.** Two saline and two non-saline gardens (fenced field plots) near the Center for Biotechnology at Borj Cedria (Tunisia). Saline 2 is the site of the original collection, while the other three sites were chosen based on the occurrence of natural populations of *Medicago truncatula* in the fall of 2008. (PDF 1 MB)

Additional file 2: **Analysis of field experiment. Mixed model results for experimental plant traits in the field experiment.** Num df: numerator degrees of freedom, Den df: denominator degrees of freedom, SO: soil origin, Pop: population, GST: garden soil type [saline/nonsaline], NS: nonsaline, S: saline. Tables to the right of each main table contain the results of post-hoc tests between each of the origin soil type by destination soil type groups. (XLS 40 KB)

Additional file 3: **Means, standard errors, and sample sizes for three phenotyping experiments.** (XLS 31 KB)

Additional file 4: **Fitness predictors in the field.** Multiple regression and single-trait linear regression to assess the relationship between aboveground biomass and pod production in naturally occurring (spontaneous) plants within our field garden exclosures. (XLS 37 KB)

Additional file 5: **Analysis of reciprocal field soil experiment.** Only plants harvested prior to greenhouse failure were included in the analysis; same patterns found when all plants included. Analyzed using mixed models with genotype and all interactions with genotype treated as random effects and significance assessed with AIC. Num df: numerator degrees of freedom, Den df: denominator degrees of freedom, SO: soil origin, Pop: population, GST: garden soil type [saline/nonsaline], LL: log-likelihood, AIC: Akaike's an information criterion. (XLS 38 KB)

Additional file 6: Analysis of 0 vs 100mM NaCl greenhouse experiment. Analyzed using mixed models with genotype and all interactions with genotype treated as random effects. Num df: numerator degrees of freedom, Den df: denominator degrees of freedom, SO: soil origin, Pop: population, GST: garden soil type [saline/nonsaline], LL: log-likelihood. Significance of random effects are χ 2 tests comparing the difference in -2 log likelihood of the full model and the model excluding that factor with 1 degree of freedom. (XLS 30 KB)

Additional file 7: **Genome-wide LD decay.** Linkage disequilibrium (r^2^ between SNPs) decay across each *M. truncatula* chromosome. Light gray denotes the 5% and 95% quantiles; dark gray denotes the 10% and 90% quantiles. (PDF 38 KB)

Additional file 8: **Genome-wide hierarchical F-statistics.** Distribution of hierarchical F-statistics computed for each SNP identified in the collection of 39 Tunisian *M. truncatula*. Note that the x-axes differ. (PDF 75 KB)

Additional file 9: **Results from 5 replicate STRUCTURE runs on biallelic SNPs in 39 Tunisian** ***M. truncatula*** **.** A) Evanno's delta K statistic, B) Likelihood scores of each run, C) representative distruct plots for K = 2 to 7. Note that delta K peaks strongly at K = 2. (PDF 92 KB)

Additional file 10: **Migrate-n parameter estimates.** Means, standard deviation, and ranges of 10 replicate runs with 96 10Kbp intervals each. Saline: S1, S2; non-saline: NS1, NS2. Two individual run results below with saline and non-saline populations with the most similar estimates of theta and with the most dissimilar estimates of theta; note the wide and overlapping confidence intervals. (XLS 41 KB)

Additional file 11: **Soil-assorting SNPs. SNPs that associate with soil type using a chi-squared test with Bonferroni correction at 0.05 family wide error rate.** Red: saline origin genotypes. Blue: non-saline origin genotypes. (XLS 66 KB)

Additional file 12: **Genes in soil-assorting LD blocks.** 198 genes contained within the 16 soil-assorting LD blocks. (XLS 44 KB)

Additional file 13: **Candidate genes.** Genes in soil-assorting LD blocks with soil-assorting amino-acid mutations. (XLS 40 KB)

Additional file 14: **Candidate amino acid substitutions.** Amino acid changes in soil-assorting genes. * indicates conserved a.a. (XLS 32 KB)

Additional File 15: **Gene trees of CPK and CIPK gene candidates.** Phylogenetic analysis of (A) CPK and (B) CIPK proteins in *M. truncatula* (red circles) and *Arabidopsis* (green circles). Maximum Likelihood trees with bootstrap support shown (1000 replications). Trees are unrooted, as these gene families have expanded as far back as mosses. Focal genes discussed in main text are depicted by filled circles. (PDF 64 KB)

Additional file 16: **False discovery rate 0.01 genes.** Genes intersected by SNPs that assort with soil type at a FDR < 0.01 threshold. (XLS 164 KB)

Additional File 17: **Using Sanger sequence data to set empirical thresholds for SNP-calling in Illumina data.** AlleleBalance (AB): For bi-allelic sites, the genotype-quality-weighted ratio of ref bases/(ref bases + alternate allele bases). The annotated value is the average over each sample with a heterozygous genotype of the (GQ value) x (the actual allele balance ratio).; BaseQualityRankSumTest (BaseQRankSum): The phred-scaled p-value from the Wilcoxon Rank Sum Test of het vs. ref base qualities.; DepthOfCoverage (DP): The depth of coverage at the given position (including spanning deletions if present).; HomopolymerRun (HRun): The length of the largest contiguous homopolymer run of the variant allele in either direction.; MappingQualityRankSumTest (MQRankSum): The phred-scaled p-value from the Wilcoxon Rank Sum Test of het vs. ref read mapping qualities.; MappingQualityZero (MQ0): The number of mapping-quality zero reads at the position.; QualByDepth (QD): The QUAL (confidence) value of the VCF record divided by the sum of depths of all samples with non-reference genotypes.; RMSMappingQuality (MQ): The root mean square mapping quality of the reads in the pileup.; SpanningDeletions (Dels): The percentage\ of reads with deletions spanning this position.; HaplotypeScore (HaplotypeScore): Estimate of the probability that the reads at this locus are coming from no more than 2 (very local) haplotypes. RED: Not a Sanger SNP (False Positive). CYAN: A Sanger SNP (True Positive). Size of point: allele frequency in the 39 TN lines. (PDF 326 KB)

Additional file 18: **Empirical threshold for SNP-calling in Tunisian** ***M. truncatula*** **Illumina data.** Four loci were sequenced in all forty TN lines in both directions (see Methods). RED: Not a Sanger SNP (False Positive). CYAN: A Sanger SNP (True Positive). Size of point: allele frequency in 40 TN lines. QD: GATK quality scaled by depth, line at QD = 30 is the selected threshold for SNP calling and the number of false positives/true positives is given for each of the four loci. (PDF 92 KB)
