# Supplementary figures and images for: The ecological genomic basis of salinity adaptation in Tunisian Medicago truncatula
Source: BMC Genomics. 2014 Dec 22;15(1):1160. doi: 10.1186/1471-2164-15-1160 (PMC4410866; doi:10.1186/1471-2164-15-1160)

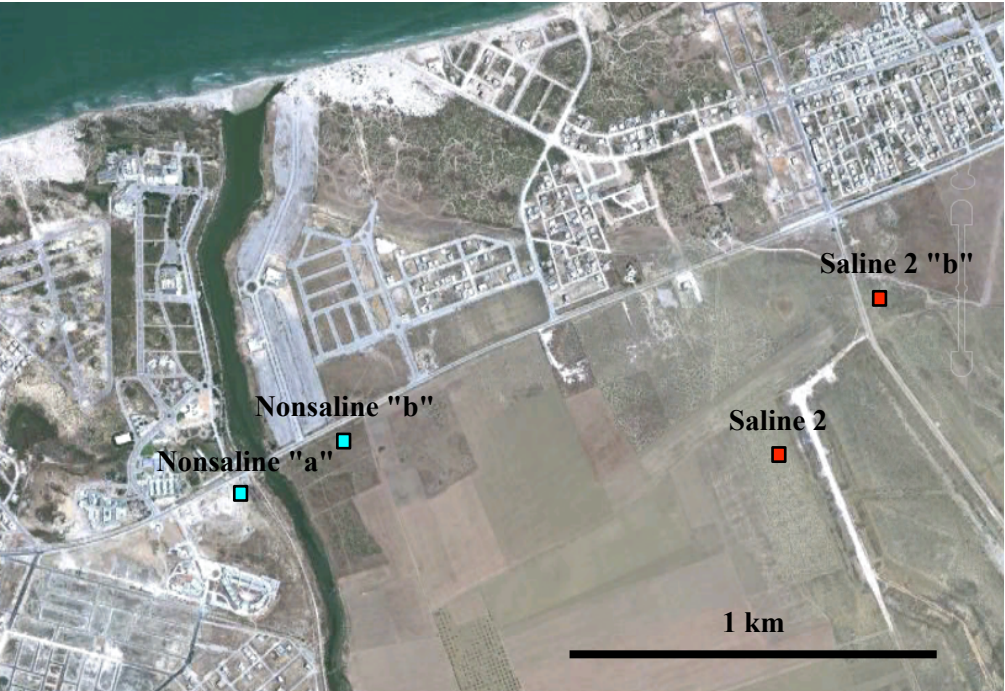

**Nonsaline "a"**

**Nonsaline "b"**

**Saline 2**

**Saline 2 "b"**

**1 km**

Supplement: Supplementary file 1 — Additional file 1: Map of field experimental sites. Two saline and two non-saline gardens (fenced field plots) near the Center for Biotechnology at Borj Cedria (Tunisia). Saline 2 is the site of the original collection, while the other three sites were chosen based on the occurrence of natural populations of Medicago truncatula in the fall of 2008. (PDF 1 MB) [file 12864_2014_6892_MOESM1_ESM.pdf]

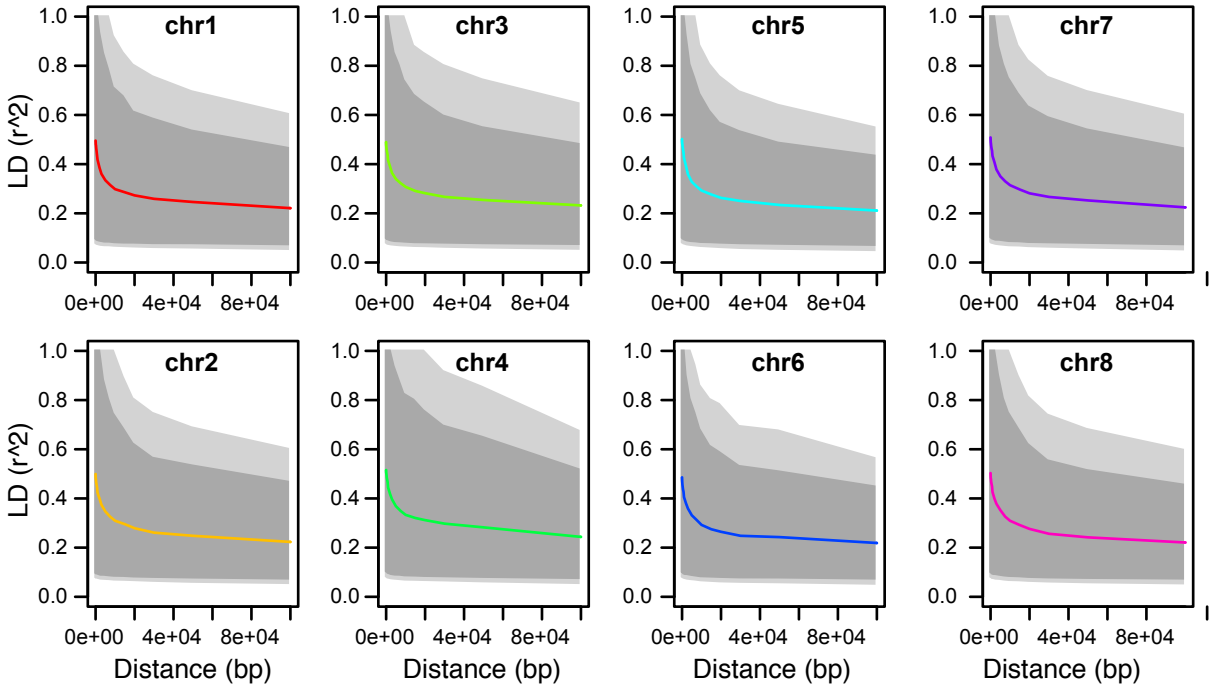

Supplement: Supplementary file 7 — Additional file 7: Genome-wide LD decay. Linkage disequilibrium (r2 between SNPs) decay across each M. truncatula chromosome. Light gray denotes the 5% and 95% quantiles; dark gray denotes the 10% and 90% quantiles. (PDF 38 KB) [file 12864_2014_6892_MOESM7_ESM.pdf]

E

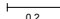

Supplement: Supplementary file 15 — Additional File 15: Gene trees of CPK and CIPK gene candidates. Phylogenetic analysis of (A) CPK and (B) CIPK proteins in M. truncatula (red circles) and Arabidopsis (green circles). Maximum Likelihood trees with bootstrap support shown (1000 replications). Trees are unrooted, as these gene families have expanded as far back as mosses. Focal genes discussed in main text are depicted by filled circles. (PDF 64 KB) [file 12864_2014_6892_MOESM15_ESM.pdf]

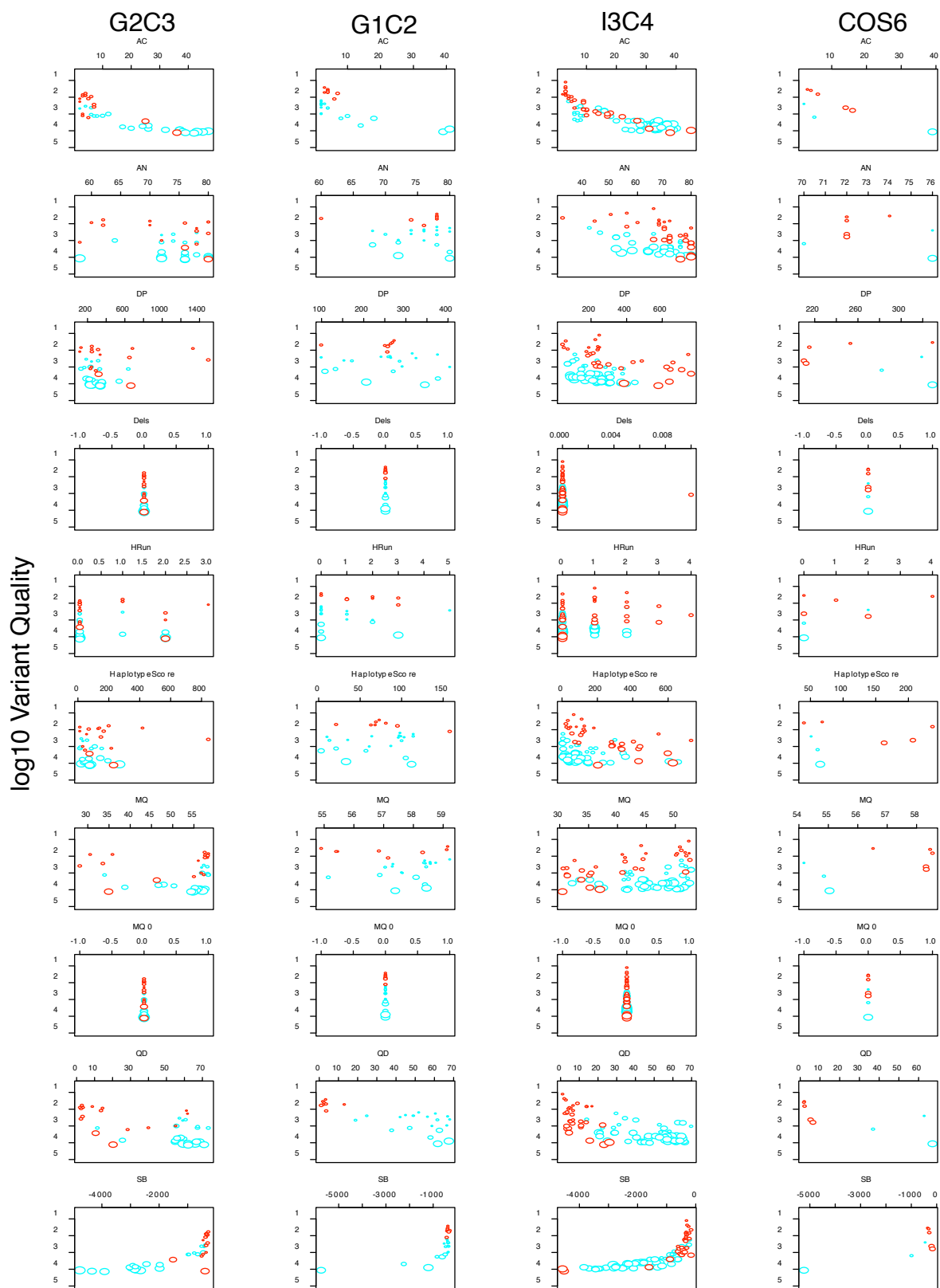

**Figure S7.**

Supplement: Supplementary file 17 — Additional File 17: Using Sanger sequence data to set empirical thresholds for SNP-calling in Illumina data. AlleleBalance (AB): For bi-allelic sites, the genotype-quality-weighted ratio of ref bases/(ref bases + alternate allele bases). The annotated value is the average over each sample with a heterozygous genotype of the (GQ value) x (the actual allele balance ratio).; BaseQualityRankSumTest (BaseQRankSum): The phred-scaled p-value from the Wilcoxon Rank Sum Test of het vs. ref base qualities.; DepthOfCoverage (DP): The depth of coverage at the given position (including spanning deletions if present).; HomopolymerRun (HRun): The length of the largest contiguous homopolymer run of the variant allele in either direction.; MappingQualityRankSumTest (MQRankSum): The phred-scaled p-value from the Wilcoxon Rank Sum Test of het vs. ref read mapping qualities.; MappingQualityZero (MQ0): The number of mapping-quality zero reads at the position.; QualByDepth (QD): The QUAL (confidence) value of the VCF record divided by the sum of depths of all samples with non-reference genotypes.; RMSMappingQuality (MQ): The root mean square mapping quality of the reads in the pileup.; SpanningDeletions (Dels): The percentage\ of reads with deletions spanning this position.; HaplotypeScore (HaplotypeScore): Estimate of the probability that the reads at this locus are coming from no more than 2 (very local) haplotypes. RED: Not a Sanger SNP (False Positive). CYAN: A Sanger SNP (True Positive). Size of point: allele frequency in the 39 TN lines. (PDF 326 KB) [file 12864_2014_6892_MOESM17_ESM.pdf]
