# Supplementary material for: The ecological genomic basis of salinity adaptation in Tunisian Medicago truncatula
Source: BMC Genomics. 2014 Dec 22;15(1):1160. doi: 10.1186/1471-2164-15-1160 (PMC4410866; doi:10.1186/1471-2164-15-1160)
Supplement: Supplementary file 8 — Additional file 8: Genome-wide hierarchical F-statistics. Distribution of hierarchical F-statistics computed for each SNP identified in the collection of 39 Tunisian M. truncatula. Note that the x-axes differ. (PDF 75 KB) [file 12864_2014_6892_MOESM8_ESM.pdf]

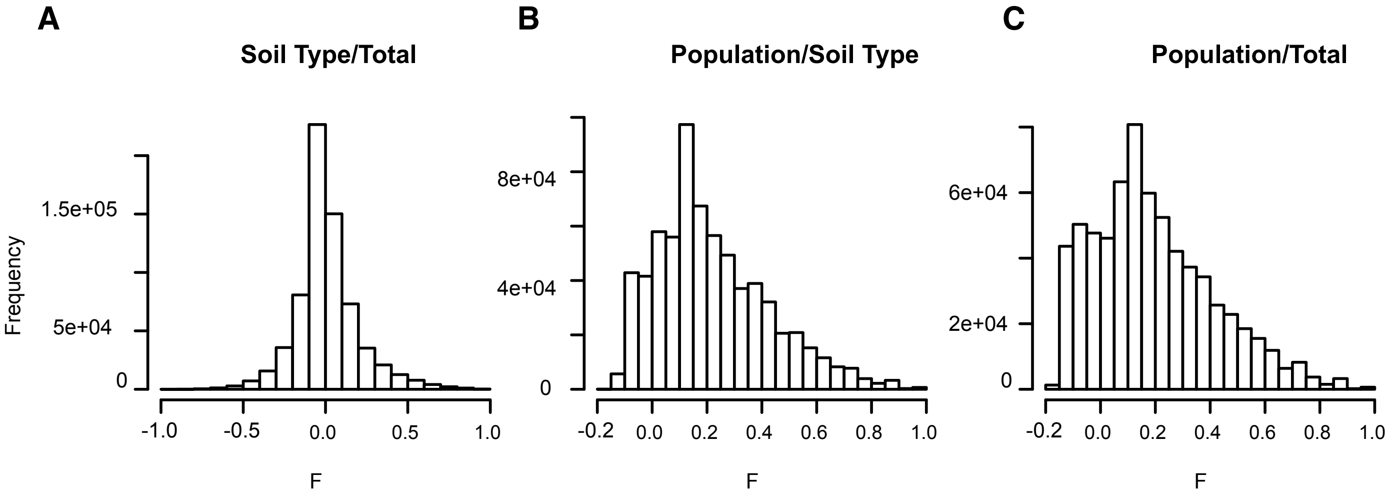

Figure S4. Hierarchical F-statistics. Distribution of hierarchical F-statistics computed for each SNP identified in the collection of 39 Tunisian *Medicago truncatula*. **C** is the classical *Fst* statistic. Note that the x-axes differ.
