# Supplementary material for: The ecological genomic basis of salinity adaptation in Tunisian Medicago truncatula
Source: BMC Genomics. 2014 Dec 22;15(1):1160. doi: 10.1186/1471-2164-15-1160 (PMC4410866; doi:10.1186/1471-2164-15-1160)
Supplement: Supplementary file 9 — Additional file 9: Results from 5 replicate STRUCTURE runs on biallelic SNPs in 39 Tunisian M. truncatula . A) Evanno's delta K statistic, B) Likelihood scores of each run, C) representative distruct plots for K = 2 to 7. Note that delta K peaks strongly at K = 2. (PDF 92 KB) [file 12864_2014_6892_MOESM9_ESM.pdf]

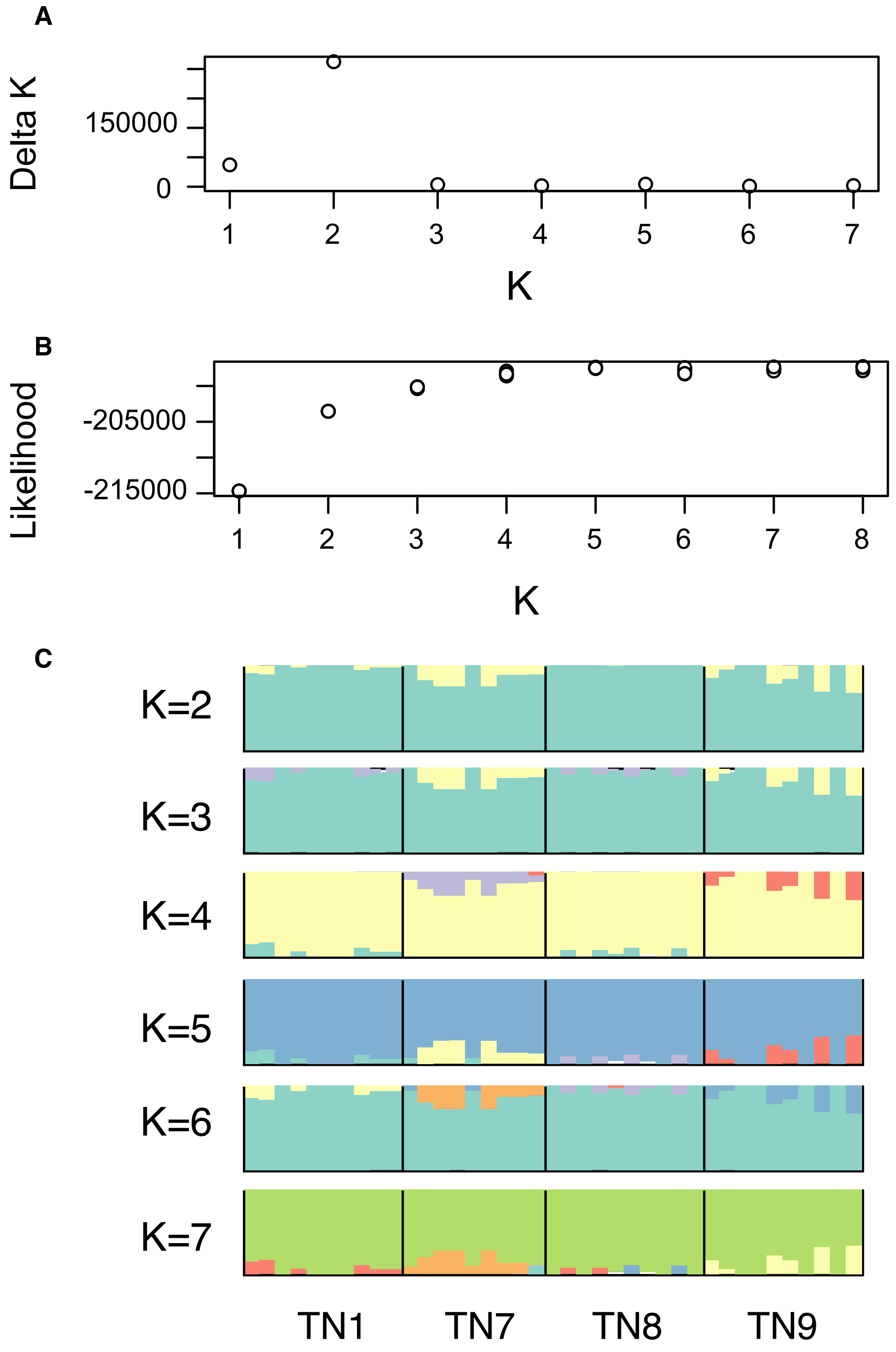

Figure S5. Results from 5 replicate STRUCTURE runs on biallelic SNPs in 39 Tunisian *Medicago truncatula*. A) Evanno's delta K statistic, B) Likelihood scores of each run, C) representative distruct plots for K = 2 to 7. Note that delta K peaks strongly at K=2.
