# Supplementary material for: The ecological genomic basis of salinity adaptation in Tunisian Medicago truncatula
Source: BMC Genomics. 2014 Dec 22;15(1):1160. doi: 10.1186/1471-2164-15-1160 (PMC4410866; doi:10.1186/1471-2164-15-1160)
Supplement: Supplementary file 18 — Additional file 18: Empirical threshold for SNP-calling in Tunisian M. truncatula Illumina data. Four loci were sequenced in all forty TN lines in both directions (see Methods). RED: Not a Sanger SNP (False Positive). CYAN: A Sanger SNP (True Positive). Size of point: allele frequency in 40 TN lines. QD: GATK quality scaled by depth, line at QD = 30 is the selected threshold for SNP calling and the number of false positives/true positives is given for each of the four loci. (PDF 92 KB) [file 12864_2014_6892_MOESM18_ESM.pdf]

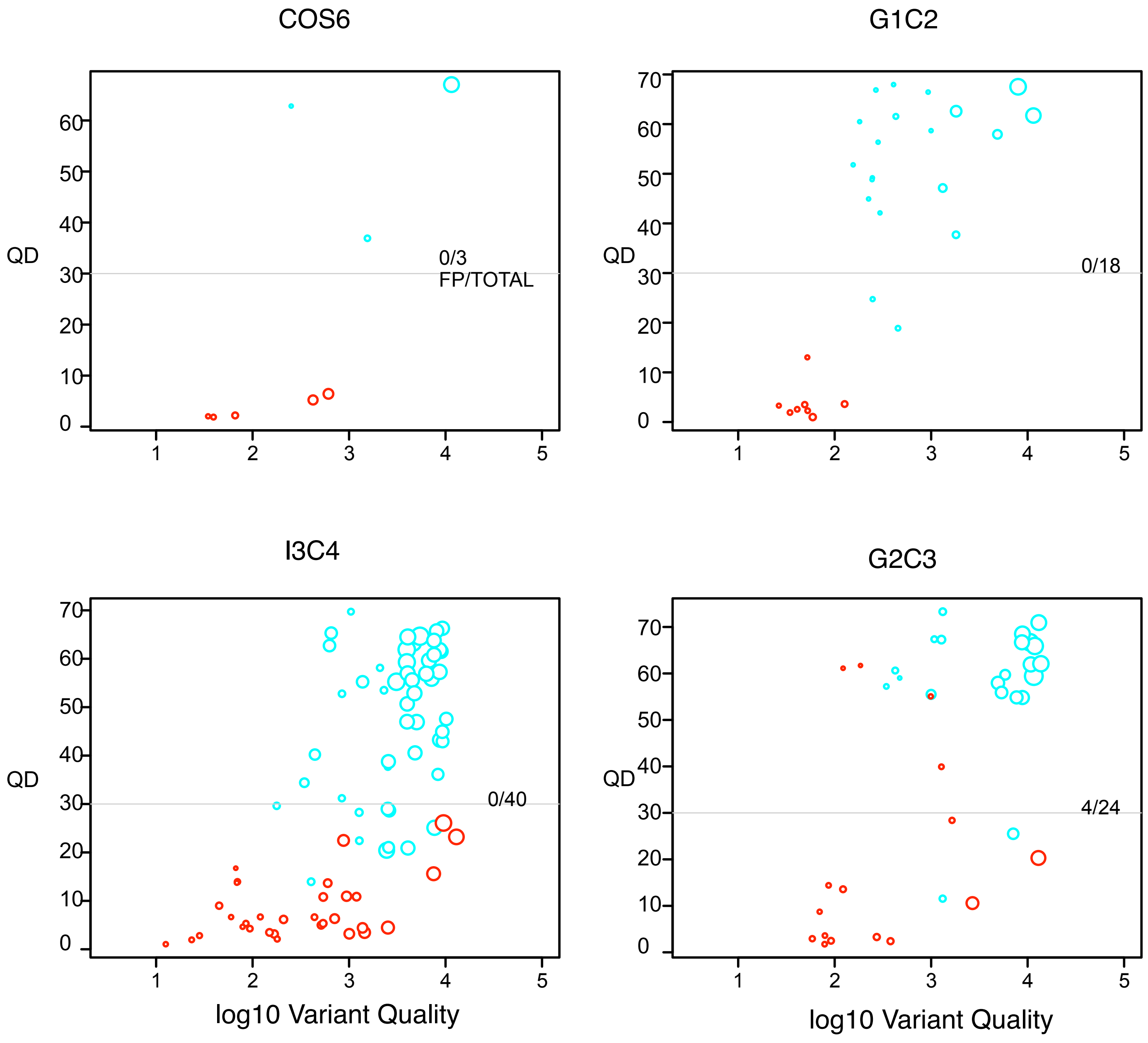

Figure S8. Sanger sequence data usedto set empirical thresholds for SNP-calling in Illumina data. Four loci were sequenced in all forty TN lines in both directions (see Methods). **RED**: Not a Sanger SNP (False Positive). **CYAN**: A Sanger SNP (True Positive). Size of point: allele frequency in 40 TN lines. QD: GATK quality scaled by depth, line at QD=30 is the selected threshold for SNP calling and the number of false positives/true positives is given for each of the four loci.
